# Body composition by dual X-ray absorptiometry (DXA) in healthy Brazilian children and adolescents: normative data

**DOI:** 10.1186/s42358-025-00479-y

**Published:** 2025-09-29

**Authors:** Melissa Mariti Fraga, Filipe Pedroso de Sousa, Gleice Clemente, Marcelo M Pinheiro, Maria Teresa Terreri

**Affiliations:** 1 Paediatric Rheumatology Unit, Paediatrics Department, Universidade Federal de São Paulo, Rua Doutor Bacelar 173, 12º andar. CEP, São Paulo, 04026-000 Brazil; 2 Medical School - Universidade Federal de São Paulo, São Paulo, Brazil; 3 Division of Rheumatology, Department of Medicine, Universidade Federal de São Paulo, São Paulo, Brazil

**Keywords:** Body composition, X-ray absorptiometry, Children, Adolescents, Lean mass, Fat mass

## Abstract

**Background:**

There is a lack of information regarding body composition (BC) measurements by Dual X-ray emission absorptiometry (DXA) in pediatric age group, mostly normative data to allow comparison among different populations and establish cut-off values. Aging itself, several chronic diseases and use of concomitant medications are associated with changes in BC, which can lead to cardiovascular events, metabolic complications, increased risk of functional loss, falls and death. Our purpose of the study is to establish BC normative data by DXA in healthy Brazilian children and adolescents.

**Methods:**

Healthy, eutrophic/overweight children and adolescents aged between 5 and 19 years were included. Medical conditions that could interfere with bone mass or BC were excluded. Participants underwent a clinical interview including details about food intake, sun exposure and physical activity. In addition, anthropometric data, pubertal stage analysis, and lumbar spine and whole body DXA measurements using Hologic QDR 4500 were performed in all participants. Individuals were divided into 4 groups: female and male with two age intervals each (between 5 and 9 years and between 10 and 19 years). BC parameters were bone mineral density (BMD), bone mineral content (BMC), total lean mass (TLM), appendicular lean mass (ALM), appendicular lean mass index (ALMI), fat mass (FM), fat mass index (FMI) and android to gynoid fat ratio (A/G) to obtain normative data according to age and gender.

**Results:**

A total of 349 healthy volunteers were eligible and enrolled for the study. Females had significantly higher values ​​of fat mass values ​​than males. All BC measurements were significantly higher in pubescent than prepubescent girls and boys.

**Conclusion:**

Our data provided BC normative data for healthy male and female Brazilian children and adolescents.

**Clinical trial number:**

Not applicable.

## Background

The assessment of body composition (BC) provides to measure the proportion of fat mass (FM), lean mass (LM) and bone mass (BM) in a 3-compartment model. Growth, aging, various chronic diseases, and some medications, such as corticosteroids, anticonvulsants, antiretrovirals, proton pump inhibitors, and anticoagulants, are associated with BC changes (decrease in LM and BM and increase in FM) [1] and higher risk for cardiovascular events, metabolic syndrome and functional loss [2].

Previous studies carried out in other countries (United States of America [3–5], Japan [6], Sweden [7], Iran [8], China [9], India [10]) and in southern Brazil [11] described that LM and BM increased with age in both sexes. Lifestyle, genetic and sociodemographic factors influence body composition and data specific to each population may avoid the risk of misclassifying children and adolescents as a low lean phenotype [11] or a low bone mineral content.

During the first years of life, there is a predominance of LM in males, which becomes significantly greater during adolescence. LM represents 22 to 25% of the body mass of a newborn and approximately 30 to 45% of the total body weight of an adult [12]. LM is influenced by several factors such as age, sex, nutrition, physical exercise, and hormonal and metabolic patterns. In boys between 5 and 17 years of age, an increase of 42 to 54% is estimated. In girls, there is an increase of 40 to 45% between 5 and 13 years of age [13]. Around 12 years of age, LM gain in girls begins to stabilize, and in boys, the gain is gradual. Total LM is stable for 15–16 years in females and 17–19 years in males, like the time of reaching adult height and bone mineral density. In adulthood, males have on average 20 kg more LM than females [14]. During the childhood growth period, the increase in BM is mainly due to an increase in bone size, with little variation in volumetric bone density [15]. Bone is a structure in constant transformation under a process of continuous formation and resorption, where genetic factors are the most important predictors of BM peak (70 to 80%) while environmental factors influence the other 20 to 30% [16]. The bone modeling process is responsible for the change in bone shape and is present from birth until the end of longitudinal growth. After this period, bone volume undergoes few changes [17]. BM doubles in the first year of life, and approximately 37% of total adult BM is formed during adolescence. The annual increase in bone mineral density (BMD) is greatest in the first 3 years of life and declines until puberty, when it increases again, accompanied by the adolescent growth spurt. Dual X-ray emission absorptiometry (DXA) is considered the gold standard method for analysis of these three compartments (FM, LM, BM) because it has good performance, accuracy, precision, feasibility, costs and low radiation dosage [18–20]. However, there are few studies for the pediatric age group concerning normative data. Due to the scarcity of normative data in Brazilian pediatric population of all ages, we performed this study to establish references in our country.

In this study, we aimed at demonstrating reference values ​​of children and adolescents from southeastern Brazil aged between 5 and 19 years for BC: FM, fat mass index (FMI), LM, appendicular lean mass (ALM), appendicular lean mass index (ALMI), bone mineral density (BMD) and bone mineral content (BMC).

## Participants and methods

This is a cross-sectional study carried out from September 2017 to January 2021.

### Participants

This study included 349 healthy children and adolescents, of which 178 were female and 171 were male. Inclusion criteria were children and adolescents aged between 5 and 19 years, healthy normal weight/overweight (Bone Mass Index - BMI Z-score between − 2 to + 2 for sex and age). BMI in children, unlike adults, is assessed in relation to age and sex specific growth curves, which are provided by the World Health Organization (WHO) [21]. Individuals with diseases or use of medications that could interfere with bone density or BC measurements, including corticosteroids, or with a history of fractures without trauma were excluded, as well as pregnant women, Indigenous Peoples, Asian and Black individuals and/or with first and second-degree relatives with low bone mass. Children or adolescents who were tall or short (Z-score for height greater than + 2 or less than − 2 SD) [21] were not eligible.

Healthy volunteers were recruited by distributing pamphlets among patients’ families and University staff, groups of university students, groups of parents of children and adolescents from various schools in São Paulo and at sports centers.

To ensure the validity and representativeness of our findings, the sample size was determined through a rigorous sample size calculation. This calculation was based on the population of children and adolescents aged 5 to 19 living in the São Paulo Metropolitan Region. We used a 95% confidence level (indicating high reliability of the results) and a 5% margin of error (defining acceptable precision). Additionally, a beta (β) of 0.20 was considered, which ensures 80% statistical power, increasing the likelihood of detecting significant differences or effects that may exist in the study population.

Participants were initially divided according to chronological age from 5 to 9 years and from 10 to 19 years, as defined by the Word Health Organization (WHO) in children and adolescents [22]. These 2 groups behave differently in relation to the speed of linear growth and body changes with the influence of sex hormones in adolescence. For data analysis, the group was again divided into 3-year intervals to have more reliable data, especially within the group of adolescents who behave differently in relation to the beginning and end of adolescence in relation to changes in body composition. Analyzing each age group individually was not feasible due to our sample’s constraints.

Data on concomitant illnesses, previous illnesses, use of medications, dietary habits (intake of foods rich in calcium), consumption of alcohol or smoking, physical activity [23]pubertal stage [24, 25] were collected.

### Methods

Weight and height were measured, and the body mass index (BMI) was calculated by dividing weight by height squared (BMI = kg/m^2^). This value was described in Z-score on the BMI graph according to the participant’s gender and age [26].

The classification of pubertal stage (Tanner Staging, also known as Sexual Maturity Rating) was self-reported and performed according to the literature [24, 25]. The Tanner Staging classification is a system used to assess pubertal development, which is divided into five stages in both sexes (females and males). These stages are based on the development of secondary sexual characteristics, such as breast growth (B1 to B5) and pubic hair development in females (P1 to P5), and genitals (G1 to G5) and pubic hair (P1 to P5) development in males [24].

Information on current intake of milk and dairy products was collected. Classification criteria for physical activity and number of hours of activity were determined according to the World Health Organization (WHO) and the International Physical Activity Questionnaire (IPAQ). Participants were considered active when they practiced physical activity for 60 min or more of moderate or intense activity per day [22, 23]. Data on alcohol consumption or smoking were collected.

BC was analyzed by DXA using a Hologic QDR 4500 device. Participants should be in supine position with arms along the body. The participants needed to be able to remain immobile in the same position during the exam for 7 to 13 min.

The parameters obtained were FM in kilograms (kg), LM in kg, ALM in kg obtained by adding the lean mass of the upper and lower limbs, BMC in grams (g), BMD in g/m^2^. Using the ALM data, the ALMI was calculated according to the model proposed by Baumgartner et al. (ALMI = ALM/height^2^) [27] and by the sarcopenia project of the Foundation for the National Institutes of Health (FNIH) Biomarkers Consortium Sarcopenia Project (ALMI_BMI_= ALM/BMI) [28]. The FMI was calculated by the ratio between the FM and the square of the height. The android/gynoid index was obtained using DXA information.

The same examiner performed all the acquisitions and analysis of the exams. The quality control of the device was performed daily using an anthropomorphic phantom, as recommended by the manufacturer. After three successive measurements on 15 volunteers on the same occasion and with repositioning, the BC accuracy values ​​expressed as root mean square coefficients of variation (CV) for FM in kg, BMD, BMC, TLM, ALM were 1.17; 0.72; 1.21; 1.18 and 1.44%, respectively [29].

For the definition of normative data, the results of body composition measurements (FM, FMI, TLM, ALMI, BMD) were used according to the grouping of the pre-defined groups: age group divided every 3 years for females and males.

Normality in data distribution was verified using the Kolmogorov-Smirnov test. Mean comparisons between two groups were performed using Student’s t test. The linear association between two numerical variables was assessed via Spearman’s correlation. To assess the effects of sex, age, and BMI (predictive variables) on ALMI, ALMI_BMI,_ TLM and FMI (dependent variables) linear regression models were used. Additionally, to assess the distinct effects of BMI and age by sex on the dependent variables, interaction effects between sex and these two variables were included. In the initial multivariate model, all predictor variables were considered. Then, non-significant interactions at 5% were eliminated. The linear regression model presents normality in the data as one of the assumptions. In this study, the Kolmogorov-Smirnov tests showed that the dependent variables ALMI, ALMI_BMI_ and FMI did not have a normal distribution, however, for sufficiently large samples, they approached normality.

For all statistical tests, a significance level of 5% was used. Statistical analyzes were performed using SPSS 20.0 and STATA 17 statistical software.

This study was approved by the Ethics Committee of the University (UNIFESP) ethics committee number 1.946.949 / CAAE 61347616.3.0000.5505 and all participants and their legal guardians read and signed the informed consent.

## Results 

Of the 349 participants, 178 were female (49.4% prepubescent and 50.5% pubertal) and 171 were male (50.3% prepubescent and 49.7% pubescent). All participants were residents of the metropolitan region of São Paulo. They declared themselves as White or Mixed-race population. We chose not to analyze the data of four volunteers who participated in the initial survey because they identified as Black (three volunteers) or Asian (one volunteer). It would not be possible to analyze this group separately.

Regarding the factors that could influence bone health, 219 (63%) were engaged in moderate to high level of physical activity (more than 1 h a day): 38 (43.3%) were female adolescents, 69 (79.3%) were female children, 47 (57.1%) were male adolescents and 65 (76.4%) were male children. None of the participants was a smoker or consumed alcohol. None of the participants received vitamin supplement such as calcium carbonate or vitamin D. Ninety-six (27%) were not in the habit of consuming milk and dairy products: 35% of pubescent girls, 23% of pubescent boys, 13% of prepubescent girls and 15% of prepubescent boys. None of the participants had a history of long bone fractures. There was no difference in relation to the reference values ​​of BC, especially LM and BM when adding overweight participants into the group. The difference was also not significant when evaluating the FM of these participants.

Table 1 shows the comparison of the clinical variables of BC according to sex. Females had higher values ​​of BMI, FM, and FMI and lower values ​​of ALMI and ALMI_BMI_ in relation to males.

**Table 1:** Comparison of clinical, anthropometric and body composition parameters according to sex

Variables	Total (*n* = 349)	Female (*n* = 178)	Male (*n* = 171)	*p*-value
Age (years)	10.9 ± 4.1	11.0 ± 4.3	10.8 ± 4.0	0.743
BMI (kg/m^2^)	19.1 ± 3.4	19.4 ± 3.3	18.8 ± 3.4	**0.017**
Weight (kg)	39.9 ± 16.3	39.6 ± 14.6	40.2 ± 18	0.365
Height (m)	1.41 ± 0.19	1.40 ± 0.17	1.42 ± 0.20	0.112
BMD (g/cm^2^)*	0.815 [0.709; 0.928]	0.805 [0.708; 0.930]	0.822 [0.709; 0.919]	0.745
BMC (g)*	1094 [854; 1642]	1097 [850; 1610]	1090 [861; 1665]	0.623
FM (kg)*	11.0 [7.3; 15.4]	12.9 [8.4; 17.2]	9.0 [6.4; 13.2]	**< 0.001**
FMI (kg/m^2^)*	5.3 [4.3; 7.3]	6.4 [5.0; 8.1]	4.4 [3.7; 6.0]	**< 0.001**
TLM (kg)	26.6 ± 11.2	24.9 ± 8.5	28.3 ± 13.2	0.131
ALM (kg)	11.7 ± 5.7	10.8 ± 4.4	12.7 ± 6.7	0.108
ALMI (kg/ m^2^)	5.5 ± 1.4	5.3 ± 1.2	5.8 ± 1.6	**0.005**
ALMI_BMI_ (kg/m^2^)	0.596 ± 0.229	0.544 ± 0.171	0.650 ± 0.266	**< 0.001**
A/G index	0.735 ± 0.134	0.734 ± 0.131	0.736 ± 0.138	0.810

BMI: body mass index; BMD: bone mineral density; BMC: bone mineral content; FM: fat mass; FMI: fat mass index; TLM: total lean mass; ALM: appendicular lean mass; ALMI: appendicular lean mass index; A/G: android/ginoid. Average ± standard deviation or median and range. * median [minimum; maximum]

In the prepubertal group, all the girls presented Tanner’s sexual development staging at B1 and P1, suggesting no development of sexual characters. None of the girls in the prepubertal group had had menarche. The same occurred in the group of prepubertal boys, where all of them presented Tanner’s sexual development staging at G1 and P1, in other words, absence of sexual characters. In the pubescent girls’ group, 65 out of 90 (72.2%) had already had menarche with a mean age of 11.3 years.

**Graph 1:**
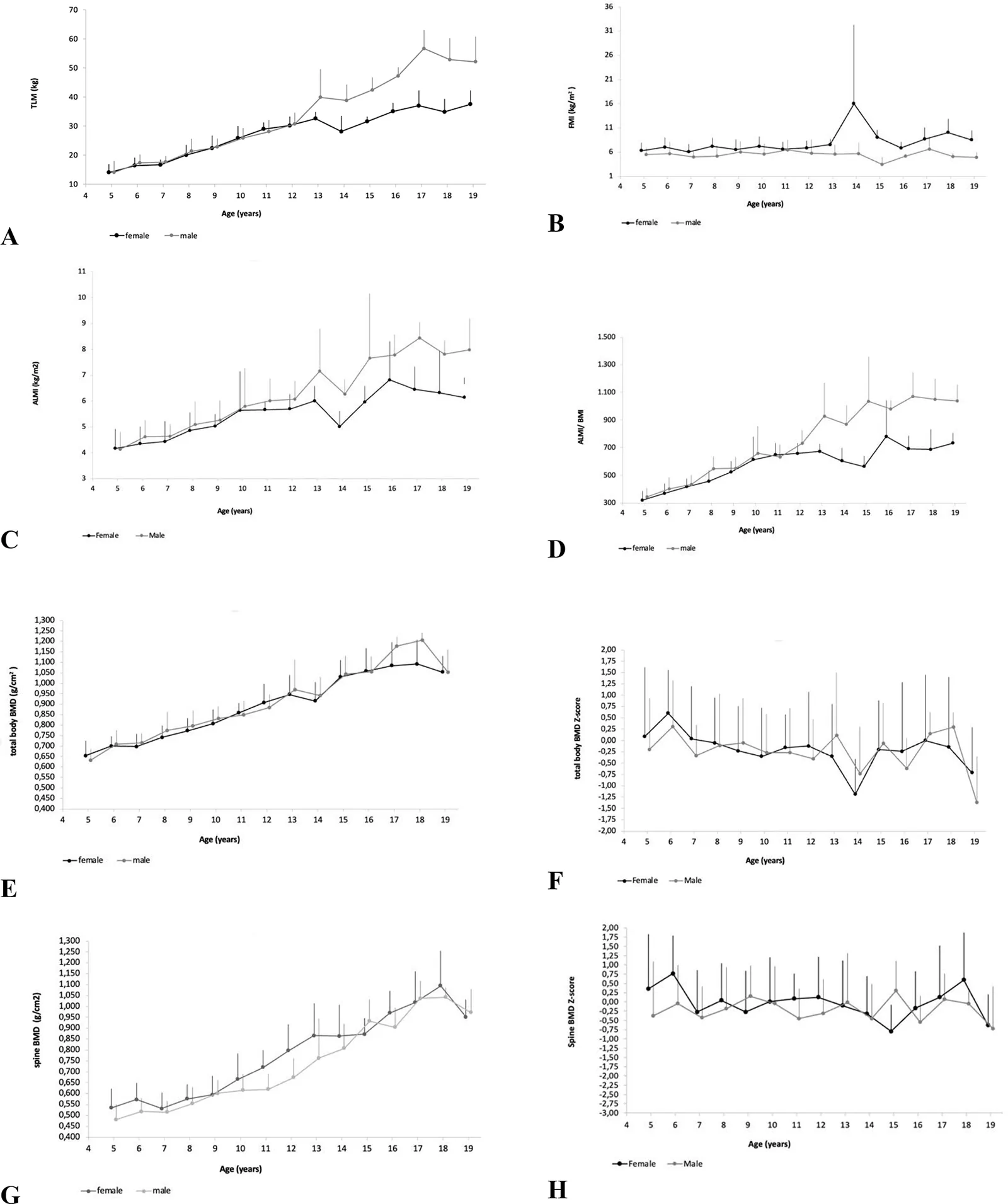
Mean and standard deviation of total lean mass (TLM) (**A**), fat mass index (FMI) (**B**), appendicular lean mass index (ALMI) (**C**), ALMI_BMI_ (**D**), total body bone mineral density (BMD) (**E**), total body BMD Z-score (**F**), spine BMD (**G**) and spine BMD Z-score (**H**) by age in males and females

The BC parameters (weight, height, BMI, TLM, ALM, ALMI, ALMI_BMI_, FM, FMI, A/G) of healthy Brazilian children and adolescents are divided by age group (intervals of 3 years) in Table 2 (female) and in Table 3 (male). Anthropometric and BC parameters were showed by median of data with minimum and maximum intervals. The normative curves of the parameters of BC are illustrated in Graph 1. For clearer visual understanding, graphs were created using participant data year by year.

**Table 2:** Clinical, anthropometric and body composition parameters of 178 healthy Brazilian girls [median (minimum: maximum)]

Age (years)	*n*	Weight (kg)	Height (m)	BMI (kg/m^2^)	TLM (kg)	ALM (kg)	ALMI (kg/m^2^)	ALMI_BMI_ (kg/m^2^)	FM (kg)	FMI (kg/m^2^)	A/G
5–7	51	24 [17; 36]	1.21 [1.04; 1.35]	17.1 [13.4; 21.3]	15.4 [9.9; 22.1]	6.2 [3.3;10.2]	4.14 [3;6.97]	0.36 [0.18; 0.55]	7.6 [4.28; 16.34]	5.56 [3.47;9.68]	0.68 [0.50;0.99]
8–10	54	35 [23;62]	1.35 [1.14;1.62]	18.7 [14.2;24.1]	22.3 [13.8;36.0]	9.6 [5.5;25.0]	5.08 [3.58;10.82]	0.51 [0.30;1.15]	11.6 [5.2;24.75]	6.28 [3.28;10.27]	0.72 [0.52;1.00]
11–13	27	49 [34;62]	1.53 [1.42;1.63]	20.4 [15.6;24.7]	30.5 [23.6; 35.9]	13.4 [10;16.2]	5.75 [4.96;6.91]	0.65 [0.53;0.79]	15.4 [7.28;23.65]	6.59 [3.24;9.29]	0.76 [0.23;0.98]
14–16	19	52 [29;67]	1.54 [1.35;1.65]	22.2 [15.9;25.0]	32.5 [19.5;40.7]	13.5 [8.3;25.3]	5.82 [4.42;10.02]	0.62 [0.47;1.36]	16.7 [8.2;27.20]	6.91 [4.11;10.28]	0.72 [0.51;0.91]
17–19	27	60 [44;81]	1.60 [1.51;1.78]	22.9 [17.7;29.1]	36.6 [26.3;43.7]	16 [10.9;23.3]	6.22 [4.02;9.12]	0.7 [0.52;0.96]	21.5 [11.7;35.50]	8.52 [5.16;13.16]	0.81 [0.58;1.01]

BMI: body mass index; TLM: total lean mass; ALM: appendicular lean mass; ALMI: appendicular lean mass index; FM: fat mass; FMI: fat mass index; A/G: android/ginoid. Median [minimum; maximum]

**Table 3:** Clinical, anthropometric and body composition parameters of 171 healthy Brazilian boys [median (minimum: maximum)]

Age (years)	*n*	Weight (kg)	Height (m)	BMI (kg/m^2^)	TLM (kg)	ALM (kg)	ALMI (kg/m^2^)	ALMI _BMI_ (kg/m^2^)	FM (kg)	FMI (kg/m^2^)	A/G
5–7	53	23 [18;40]	1.20 [1.08;1.40]	16.22 [13.44;24.04]	16.5 [1.5;24.1]	6.5 [3.8;10.1]	4.40 [3.24;5.79]	0.38 [0.24;0.66]	6 [3.3;17.8]	4.36 [2.63;10.71]	0.65 [0.42; 1.09]
8–10	48	32 [22;55]	1.37 [1.23;1.57]	17.15 [13.8;25.1]	23 [14.3;31.3]	10 [5.7;21.2]	5.22 [3.78;10.27]	0.57 [0.39;1.22]	8 [4.6;23.8]	4.43 [2.78;10.87]	0.70 [0.5;0.98]
11–13	33	44 [31;81]	1.52 [1.36;1.79]	19 [14.7;27.1]	31.2 [21.1;57.1]	13.9 [9.1;22.3]	6.44 [4.63;10.73]	0.71 [0.49;1.44]	11 [6.2;28.8]	4.67 [2.63;11.49]	0.74 [0.56–1.21]
14–16	19	58 [48;76]	1.68 [1.58;1.75]	21.1 [16;25.9]	43.38 [32.4;51.4]	20.5 [14.6;30.8]	6.94 [5.07;11.31]	0.95 [0.65;1.58]	11,7 [7.7;24.9]	4.39 [2.54;8.54]	0.78 [0.59;1.1]
17–19	18	72 [54;99]	1.75 [1.56;1.90]	23.6 [16.6;28.9]	54.1 [40.9;63.8]	24.7 [18.4;30.2]	8.09 [6.18;9.64]	1.04 [0.80; 1.29]	14 [7.9;32.5]	4.49 [2.45;9.51]	0.83 [0.6;1.13]

BMI: body mass index; TLM: total lean mass; ALM: appendicular lean mass; ALMI: appendicular lean mass index; FM: fat mass; FMI: fat mass index; A/G: android/ginoid. Median [minimum; maximum]

## Discussion

This is the first Brazilian study to perform normative curves for BC parameters, including FM, FMI, TLM, ALM, ALMI, BMC and BMD, in a pediatric population, aged between 5 and 19 years, through DXA.

The standardization for each population is important because the BC is influenced by several factors that differ among races, ethnicity, cultural aspects, eating and physical activity habits and others. In a previous study carried out with adolescents between 12 and 17 years old, in southern Brazil [11], the TLM was standardized. The southern Brazilian study with 541 adolescents (68,8% boys) showed sex difference in growth trajectories for absolute and adjusted LM, with boys presenting greater LM quantity with advancing ages than girls (66.9% and 17.4% difference between the ages of 12 and 17 for boys and girls, respectively). It was suggested that the lower percentiles (p3 and p10) could be used as an indication of a low TLM phenotype in children since there is no cut-off value as in adults. Low TLM in adults is related to metabolic diseases. In the pediatric population, these data are still controversial and the relationship between low TLM and development of metabolic diseases in childhood is still unclear [30].

In the pediatric population, both male and female, BC parameters increase with age as the organism is growing. In adolescence, boys show a significant increase in TLM while girls maintain continuous gain, but with a significant increase in FM in adolescence [31]. As we expected, FMI is higher in the female group. In our study, we observed that male participants showed greater TLM gain when compared to female participants, especially after 12 years of age. Other studies found similar results that could be explained mainly by the hormonal influence [32, 33]. In this phase, there is greater activation of growth hormone and sex steroids, which lead to a rapid increase in skeletal muscle and consequently in TLM [34]. However, we observed a slight decrease in TLM and ALMI in 14-year-old girls and boys, which would not be explained if it were not supposed to be the smaller number of participants included in this age group.

Some studies have evaluated calcium consumption or supplementation in relation to BMD and future fracture reduction [16, 35]. Also in relation to BMD, physical activity with certain characteristics appears to be better than others in improving gain [36]; Dynamic [37] and vigorous activities [38, 39] with impact and load have an important and consistent positive effect on bone development. In our study, it was not possible to evaluate the differences between diets/consumption of milk and dairy products or moderate to intense physical activity in BC. We observed that the minority of adolescents consumed milk or dairy products in their diet, unlike the children who had daily consumption. Regarding physical activity, we found participants who performed intense physical activity almost daily and others with light physical activity. Due to the size of the sample, it was not possible to evaluate individual differences.

Our study has several strengths, including BC normative data for Brazilian children and adolescents, according to sex and gender, allowing comparative analysis with other chronic medical conditions and early non-pharmacological and pharmacological therapeutic intervention measures to achieve healthier development and growth with less risk of future disease and morbidity.

On the other hand, it has some limitations. The first one refers to the impossibility of performing an intimate physical examination to assess the pubertal stage of the volunteers, using the self-reported classification by Tanner [24, 25]. Another one was that all exams were performed in a single center from the same metropolitan city. Thus, the present study may not represent the entire Brazilian pediatric population, as we are in a country of continental dimensions (although the city of São Paulo is recognized for its high miscegenation from all regions of the country). Finally, the power of the sample was not sufficient to calculate percentiles, and obese individuals were not included.

In conclusion, in this study we present normative data according to sex and age of Brazilian children and adolescents for FM, FMI, TLM, ALM, ALMI, ALMI_BMI_. Although normative curves already exist for several populations, this study serves as a database for national reference. With this data we can start to promote interventions in childhood that can prevent chronic diseases and reduce morbidity in adult life.

## Data Availability

No datasets were generated or analysed during the current study.

## Abbreviations

A/G: Android to gynoid fat ratio
ALM: Appendicular lean mass
ALMI: Appendicular lean mass index
BC: Body composition
BM: Bone mass
BMC: Bone mineral content
BMD: Bone mineral density
BMI: Body mass index
CV: Coefficients of variation
DXA: Dual X-ray emission absorptiometry
FM: Fat mass
FMI: Fat mass index
FNIH: Foundation for the national institutes of health
g: Grams
IPAQ: International physical activity questionnaire
kg: Kilograms
LM: Lean mass
M: Meter
SD: Standard deviation
TLM: Total lean mass
WHO: World health organization
